# Iron deficiency treatment pathway in Italy: patients’ perceptions of diagnosis and treatment

**DOI:** 10.57264/cer-2025-0011

**Published:** 2025-11-28

**Authors:** Ovidio Brignoli, Rose-Marie Carneiro, Cécile Fournier, Ettore Pinardi, Maurizio Serati

**Affiliations:** 1 Italian College of General Practitioners & Primary Care, Florence, Italy; 2 Patient View Department, APLUSA Research, Lyon, France; 3 Medical Department, Pierre Fabre Group, Lavaur, France; 4 Department of Obstetrics & Gynecology Urogynecology Unit, University of Insubria, Varese, Italy

**Keywords:** anemia, care pathway, iron deficiencies, patients, qualitative evaluation, quantitative evaluation, surveys

## Abstract

**Aim:**

This 2023 online survey assessed the management of patients with iron deficiency anemia/iron deficiency without anemia (IDA/IDWA) in Italy.

**Materials & methods:**

The study used the patient’s perspective to examine care pathways used to manage IDA/IDWA in Italy, to raise clinician awareness, improve patient care (earlier diagnosis, initiate appropriate treatment) and outcomes (reduce recurrence/progression). The survey questioned iron deficiency (ID) diagnoses of patients, their sources of information and influence, their knowledge of ID, perceptions and fears surrounding treatment, unmet needs and expectations, and underlying causes of treatment compliance and persistence.

**Results:**

Of the 404 respondents (102 males and 302 females) who completed the survey, all were aged between 18 and 80 years. Almost all respondents (97.0%) experienced ID symptoms, most frequently fatigue, weakness and tiredness (71.0% of the total cohort; 80.1% females vs 44.1% males); 76.8% of all respondents regarded symptoms as ‘bothersome’. Most respondents (70.5%) consulted a physician as the first action to treat ID (79.8% females vs 43.1% males); general practitioners were the main healthcare providers, consulted by 55.6% of all respondents. Most respondents (94.1%) were aware of potential ID recurrence, and 75.0% reported recurrent ID episodes since their diagnosis. Satisfaction with ID treatment was rated as average (7–7.7/10); 56.4% of all respondents stayed on ID treatment for as long as prescribed, and 41.1% did not fully comply with tablet intake, primarily because they ‘felt much better’ (64.8%).

**Conclusion:**

This survey identified high rates of self-reported ID symptoms and recurrent ID episodes among respondents. It highlights the importance of increasing awareness of ID and its consequences among healthcare practitioners and individuals in the general population, of shortening the time before diagnosis, and ensuring that patients continue treatment for the prescribed duration to resaturate serum iron levels.

## Plain language summary

**What is this article about?:**

Iron deficiency (ID) is a condition in which a person does not have enough iron in their body to maintain adequate levels of healthy red blood cells, for reasons such as heavy menstrual bleeding, low dietary iron intake or poor iron absorption. ID often leads to anemia, when there are not enough healthy red blood cells carrying oxygen throughout the body. Iron deficiency anemia (IDA) is very common, as is iron deficiency without anemia (IDWA). Untreated IDA can lead to severe health problems, including extreme fatigue, weakness, hair loss and rapid heart rate, so the proper management of IDA is important. Diagnosing IDA and IDWA is complicated by inadequate screening recommendations, a lack of awareness of IDWA among healthcare practitioners (HCPs), and the clinical signs and symptoms of ID are often ignored. HCPs also differ widely in their choice of iron compounds for managing IDA and IDWA. This paper describes findings from a survey among adults in Italy diagnosed with IDA or IDWA and treated in the past 12 months with prescription medication.

**What were the results?:**

Most survey respondents did not know their ID status prior to diagnosis and after receiving an ID treatment the main reason for no compliance was because the patient felt better.

**Why is this important?:**

The findings suggest that both HCPs and the general population need to be more aware of ID and its consequences, and how to manage ID. HCPs can play a vital role in educating and supporting patients on ID treatment, to improve their understanding about managing their disease and continuing treatment for as long as is needed.

## Shareable abstract

A survey in 404 Italian patients with iron deficiency shows that almost half do not take their oral iron treatment as prescribed. Yet 75% have recurrent episodes of iron deficiency.

#iron deficiency #anemia #iron treatment #patient perspectives #compliance

Iron deficiency (ID), the depletion of total-body iron, is a major precipitant of anemia [1,2]. According to the WHO, iron deficiency anemia (IDA) is the most common nutritional deficiency worldwide, affecting approximately a third of the global population [3], while iron deficiency without anemia (IDWA) affects at least twice as many people [1,2,3].

IDA can have profound impacts at all ages: impaired cognitive functioning in young children and adults, and cognitive decline in the elderly; reduced physical and working capacities in adults; and increased maternal morbidity and high risk of poor pregnancy outcomes [1,2]. In adult women, ID has been linked to sexual dysfunction, and a negative impact on quality of life [4,5]. Thus, adequate and appropriate management of IDA has important long-term consequences in the general population and in the clinical setting.

Clinicians are advised to treat ID regardless of whether or not anemia is involved [1]. Wide variability has been reported between clinicians in the management of IDA and IDWA, with the choice of iron compound depending upon several factors such as patient preference, clinical status (age, sex, recent onset vs chronic anemia), response to prior therapy and the goal of treatment [2,3]. To the best of our knowledge, real-world data on the management of patients with IDA/IDWA in Italy are limited to a single analysis of primary care clinical records from an Italian national database. This study reported that ID was undertreated and treatment adherence poor [6]. Surveys among patients with IDA or IDWA have indicated that many face long delays between symptom onset and diagnosis, and are not always satisfied with their treatment [7,8].

We therefore conducted a survey study to understand and describe, from the patient’s perspective, the care pathways used to manage IDA/IDWA in Italy, with the aim of raising clinician awareness, in order to potentially improve both patient care (earlier diagnosis, initiating appropriate treatment) and outcomes (reduced recurrence/progression). We collected data on how, when and by whom they were diagnosed, their sources of information and influence, their knowledge of the disease and perceptions and fears surrounding treatment, any treatment-related concerns, unmet needs and expectations and underlying causes of treatment compliance and persistence.

## Materials & methods

### Study design & participants

The survey was a 20-minute online quantitative survey of 55 questions (12 preliminary/screening questions and 43 survey questions), hosted on a secure server (Supplementary Appendix 1, translated from Italian). It was designed by APLUSA based on results from exploratory qualitative interviews conducted beforehand in 18 adult individuals with healthcare practitioner (HCP)-diagnosed IDA or IDWA (not reported here), with final approval of the survey structure from Pierre Fabre (France). Individuals were recruited by email from two online panel databases of Italian consumers. Individuals accepting the email invitation to participate were provided a link to the online survey with a unique code per respondent.

Inclusion criteria were: male or female respondents who had been diagnosed with IDA or IDWA by an HCP in the past 2 years, had been treated in the past 12 months with prescription medication for IDA or IDWA, and who provided informed consent. Predefined quotas for sex and/or age were specified as follows: ∼75% female respondents, ≤40 pregnant women, a balanced distribution across age groups for females (≥18 to ≤80 years), and only men aged 26–80-years. The first 12 questions were screening questions to assess eligibility for study inclusion, followed by 43 questions about a respondent's ID status.

If respondents met the study criteria, they proceeded to complete the survey; those who did not satisfy inclusion criteria did not proceed any further.

### Collection of study data

Participants' responses to the questionnaire were the only data collected during the survey; no other investigations were performed. The data collection confirmed the following details: sex (male or female), age between 18 and 80 years, pregnancy status, number and age of any children (<18 years); health status over the past 2 years, whether respondents had been diagnosed with IDA or IDWA by an HCP in the past 2 years, had been treated in the past 12 months with prescription medication for IDA or IDWA; number of episodes of IDA or IDWA experienced in the past 2 years (1, 2–3, 4–6, or ≥6); brand awareness of all products used to treat ID or IDWA; experience of and satisfaction with prescribed treatments; challenges surrounding treatment compliance (e.g., information, convenience, tolerability, efficacy); perceptions surrounding what tools or information could help with taking ID treatment; and sociodemographic details. Treatment satisfaction and treatment perception were graded from 1 to 10, where 1 was the worst score and 10 represented the best score.

### Ethics

Before starting the survey, participants were asked to consent to sharing medical and health information about themselves, including safety reporting or product quality issues. If they did not consent, the survey was stopped. Participants’ data were processed in a general and anonymized manner for the purposes of this study only, and were not provided to any third party. Data processed was in accordance with the General Data Protection Regulation n°2016/679 and the French Data Protection Act of 6 January 1978 on personal data. APLUSA and the study sponsor agreed to store all personal data collected from participants in a pseudonymized form for a period of 5 years after the end of the study. As this was not an investigation of clinical outcomes with a particular intervention, neither Ethics Committee approval nor clinical trial registration was required. Only descriptive summary statistics were used (totals, percentages); no formal statistical comparisons or analyses were planned.

## Results

The online survey was conducted from 30 March 2023 to 26 April 2023, with supplemental data gathered between 2 May 2023 and 27 May 2023.

Results for a total of 404 respondents were available for analysis.

### Profile of study participants

The study sample contained 74.8% female participants and 25.2% male participants (Table 1). Almost two-thirds of females (61.6%) and males (63.7%) were aged between 26 and 54 years; whereas only 16.6% of females were aged 55–80 years versus 36.3% of males.

**Table 1.** Characteristics of all 404 individuals with iron deficiency or iron deficiency anemia who participated in an online quantitative study.

	All study participants (n = 404)
Male	n = 102 (25.2)
Age^†^, years	
26–44	26 (25.5)
45–54	39 (38.2)
55–80	37 (36.3)
Female	n = 302 (74.8)
Age^†^, years	
18–25	66 (21.8)
26–44	95 (31.5)
45–54	91 (30.1)
55–80	50 (16.6)
Pregnant, yes	33 (10.9)
Geographical region of residence in Italy	n = 404
Northeast	97 (24.0)
Northwest	88 (21.8)
Central	96 (23.8)
South	72 (17.8)
Islands	51 (12.6)
Area of residence	n = 404
Urban (within city limits)	249 (61.6)
Suburban (outside of city limits)	58 (14.4)
Rural	53 (13.1)
Small city/town	44 (10.9)
Current employment status	n = 404
Full-time	226 (55.9)
Part-time (<32 h/week)	55 (13.6)
Stays home to care for family	48 (11.9)
Unemployed/seeking work	31 (7.7)
Retired/disabled/not working and not seeking work	26 (6.4)
Student/at school	17 (4.2)
Nonpaid work (e.g., volunteer, charity)	1 (0.2)
Current health status	n = 403
Very good	52 (12.9)
Rather good	239 (59.3)
Neither good nor poor	87 (21.6)
Rather poor	22 (5.5)
Very poor	3 (0.7)
Monthly income, Euro	n = 404
<499	32 (7.9)
500–1499	80 (19.8)
1500–2499	101 (25.0)
2500–3499	58 (14.4)
3500–4499	67 (16.6)
4500–5499	30 (7.4)
>5500	12 (3.0)
Did not want to respond	24 (5.9)

All data are reported as numbers and percentages of patients.

† Recruited according to predefined quotas.

### Care pathway

#### Symptoms & diagnosis of ID

In this survey, a higher proportion of males (especially those aged 26–44 years) than females discovered their ID status after experiencing symptoms (59.8 vs 38.4%), whereas a higher proportion of females compared with males discovered their ID status via a routine check-up (43.4 vs 34.3%, respectively) (Table 2). Almost all respondents (97.0%) experienced ID symptoms. The most common symptom was fatigue/weakness/tiredness, which was reported by 71.0% of the total population, but more often by females than males (80.1 vs 44.1%; p < 0.05). This was also the symptom that most commonly led patients to seek help with ID management. Overall, females reported many more symptoms than males (Table 2), whereas age was not a strong discriminant variable (except for symptoms of hair loss and heart palpitations; data not shown).

**Table 2.** Impact of sex upon the patient journey of iron deficiency discovery, first iron deficiency symptoms, and symptoms triggering treatment.

	All study participants (n = 404)	Males (n = 102)	Females (n = 302)
ID discovery	n = 404	n = 102	n = 302
After experiencing symptoms	177 (43.8)	61 (59.8)	116 (38.4)
At routine check-up	166 (41.1)	35 (34.3)	131 (43.4)
Pregnancy blood tests	N/A	N/A	22 (7.3)
At hospital admission, because the respondent felt unwell	23 (5.7)	3 (2.9)	20 (6.6)
Other	16 (4.0)	3 (2.9)	13 (4.3)
First symptoms of ID experienced	n = 404	n = 102	n = 302
Fatigue/weakness/tiredness	286 (71.0)	45 (44.1)	242 (80.1)^‡^
Headaches	198 (49.0)	52 (51.0)	146 (48.3)
Paleness	139 (34.4)	23 (22.5)	116 (38.4)^‡^
Irritability or depressive mood	139 (34.4)	27 (26.5)	112 (37.1)
Difficulty with concentration	136 (33.7)	19 (18.6)	117 (38.7)^‡^
Hair loss	120 (29.7)	29 (28.4)	91 (30.1)
Heart palpitations	111 (27.5)	14 (13.7)	97 (32.1)^‡^
Shortness of breath	100 (24.8)	27 (26.5)	73 (24.2)
GI disturbances	72 (17.8)	9 (8.8)	63 (20.9)^‡^
Other^†^	5 (1.2)	0	5 (1.7)
No symptoms	12 (3.0)	5 (4.9)	7 (2.3)
Symptoms triggering ID management	n = 392	n = 97	n = 295
Fatigue/weakness/tiredness	265 (67.6)	42 (43.3)	223 (75.6)^‡^
Headaches	104 (26.5)	31 (32.0)	73 (24.7)
Paleness	66 (16.8)	11 (11.3)	55 (18.6)
Irritability or depressive mood	57 (14.5)	14 (14.4)	43 (14.6)
Difficulty with concentration	41 (10.5)	9 (9.3)	32 (10.8)
Hair loss	57 (14.5)	17 (17.5)	40 (13.6)
Heart palpitations^‡^	50 (12.8)	5 (5.2)	45 (15.3)
Shortness of breath	42 (10.7)	12 (12.4)	30 (10.2)
GI disturbances	21 (5.4)	5 (5.2)	16 (5.4)
Other^†^	5 (1.3)	0	5 (1.7)
No symptoms	0	0	0

All data are reported as numbers and percentages of patients.

† “Other” included leg pain.

‡ p < 0.05.

GI: Gastrointestinal; GP: General practitioner; HCP: Healthcare practitioner; ID: Iron deficiency; N/A: Not available.

#### First action taken to treat ID

Analysis of the first action taken to treat ID revealed that respondents most frequently consulted a physician (general practitioner [GP] or specialist; 70.5% of respondents), with many more females than males taking this initial step (79.8 vs 43.1%; p < 0.05). More males than females sought web-based information, consulted family, friends or colleagues and visited the pharmacy (Table 3). The time taken between the first experience of symptoms and the initial consultation was less than 3 months for over two-thirds (68.3%) of all respondents; almost double the proportion of females than males had an initial consultation within 1 month of experiencing symptoms (29.5 vs 16.7%, respectively).

**Table 3.** Impact of sex upon the patient journey of the first action taken to treat iron deficiency, time between first symptoms and initiation consultation, and involvement of a healthcare practitioner.

	All study participants (n = 404)	Males (n = 102)	Females (n = 302)
First action taken to treat ID	n = 404	n = 102	n = 302
Physician consulted (GP or specialist)	285 (70.5)	44 (43.1)	241 (79.8)^¶^
Sought Internet information	42 (10.4)	24 (23.5)	18 (6.0)
Sought advice from family/relatives/friends/colleagues	37 (9.2)	21 (20.6)	16 (5.3)
Sought advice from pharmacy	12 (3.0)	6 (5.9)	6 (2.0)
Sought advice from paramedical professional (physiotherapist, nurse, midwife)	13 (3.2)	5 (4.9)	8 (2.6)
Sought advice from a specialized store (food supplements, phytotherapy stores, other)	6 (1.5)	1 (1.0)	5 (1.7)
Directly purchased a product	5 (1.2)	0	5 (1.7)
Lifestyle/diet adjusted	4 (1.0)	1 (1.0)	3 (1.0)
Time between first symptoms and initial consultation	n = 404	n = 102	n = 302
Less than 1 month	106 (26.2)	17 (16.7)	89 (29.5)
Between 1 and 3 months	170 (42.1)	48 (47.1)	122 (40.4)
Between 4 and 6 months	73 (18.1)	27 (26.5)	46 (15.2)
Between 7 and 12 months	21 (5.2)	6 (5.9)	15 (5.0)
Over 12 months	34 (8.4)	4 (3.9)	30 (9.9)
First HCP consulted for treating ID^†^	n = 404	n = 102	n = 302
GP	233 (57.7)	26 (25.5)	207 (68.5)^¶^
Gastroenterologist	13 (3.2)	8 (7.8)	5 (1.7)
Hematologist	80 (19.8)	40 (39.2)^¶^	40 (13.2)
Gynecologist	45 (11.1)	10 (9.8)	35 (11.6)
Oncologist	6 (1.5)	5 (4.9)	1 (0.3)
Nephrologist	2 (0.5)	2 (3.9)	0
Dietician	13 (3.2)	4 (3.9)	9 (3.0)
Midwife	1 (0.2)	1 (1.0)	0
Nurse	0	0	0
Pharmacist	8 (2.0)	6 (5.9)	2 (0.7)
Another specialist	0	0	
Not referred to any HCP	N/A	N/A	N/A
Do not know/cannot remember	1 (0.2)	0	1 (0.3)
HCP referred to for further exams after the first HCP consultation	n = 404	n = 102	n = 302
GP	62 (15.3)	14 (13.7)	48 (15.9)
Gastroenterologist	33 (8.2)	19 (18.6)^¶^	14 (4.6)
Hematologist	62 (15.3)	22 (21.6)	40 (13.2)
Gynecologist	44 (10.9)	8 (7.8)	36 (11.9)
Oncologist	11 (2.7)	8 (7.8)	3 (1.0)
Nephrologist	8 (2.0)	2 (2.0)	6 (2.0)
Dietician	19 (4.7)	9 (8.8)	10 (3.3)
Midwife	1 (0.2)	0	1 (0.3)
Nurse	3 (0.7)	1 (1.0)	2 (0.7)
Pharmacist	15 (3.7)	4 (3.9)	11 (3.6)
Another specialist	4 (1.0)	0	4 (1.3)
Not referred to any HCP	131 (32.4)	13 (12.7)	118 (39.1)^¶^
Do not know/cannot remember	11 (2.7)	2 (2.0)	9 (3.0)
HCP who diagnosed ID	n = 404	n = 102	n = 302
GP	201 (49.8)	26 (25.5)	175 (57.9)^¶^
Gastroenterologist	24 (5.9)	14 (13.7)	10 (3.3)
Hematologist	102 (25.2)	36 (35.3)	66 (21.9)
Gynecologist	35 (8.7)	9 (8.8)	26 (8.6)
Oncologist	6 (1.5)	5 (4.9)	1 (0.3)
Nephrologist	3 (0.7)	1 (1.0)	2 (0.7)
Dietician	11 (2.7)	5 (4.9)	6 (2.0)
Midwife	1 (0.2)	0	1 (0.3)
Nurse	4 (1.0)	1 (1.0)	3 (1.0)
Pharmacist	6 (1.5)	3 (2.9)	3 (1.0)
Another specialist	7 (1.7)	1 (1.0)	6 (2.0)
Not referred to any HCP	N/A	N/A	N/A
Do not know/cannot remember	3 (0.7)	0	3 (1.0)
HCP in charge of treatment initiation	n = 404	n = 102	n = 302
GP	229 (56.7)	37 (36.3)	192 (63.6)^¶^
Gastroenterologist	27 (6.7)	14 (13.7)	13 (4.3)
Hematologist	109 (27.0)	47 (46.1)^¶^	62 (20.5)
Gynecologist	26 (6.4)	1 (1.0)	25 (8.3)
Oncologist	1 (0.2)	1 (1.0)	0
Nephrologist	2 (0.5)	1 (1.0)	1 (0.3)
Another specialist^‡^	4 (1.0)	0	4 (1.3)
Do not know/cannot remember	6 (1.5)	1 (1.0)	5 (1.7)
HCP currently seen for treating ID	n = 297	n = 80	n = 217
GP	165 (55.6)	22 (27.5)	143 (65.9)^¶^
Gastroenterologist	20 (6.7)	10 (12.5)	10 (4.6)
Hematologist	72 (24.2)	34 (42.5)^¶^	38 (17.5)
Gynecologist	11 (3.7)	2 (2.5)	9 (4.1)
Oncologist	5 (1.7)	4 (5.0)	1 (0.5)
Nephrologist	1 (0.3)	1 (1.3)	0
Dietician	10 (3.4)	6 (7.5)	4 (1.8)
Another specialist^§^	2 (0.7)	0	2 (0.9)
No longer consulting an HCP	11 (3.7)	1 (1.3)	10 (4.6)
Do not know/cannot remember	0	0	0
HCP responsible for ID treatment prescription	n = 404	n = 102	n = 302
GP	230 (56.9)	36 (34.3)	194 (64.2)^¶^
Gastroenterologist	30 (7.4)	19 (18.6)	11 (3.6)
Hematologist	97 (24.0)	40 (39.2)^¶^	57 (18.9)
Gynecologist	30 (7.4)	2 (2.0)	28 (9.3)
Oncologist	3 (0.7)	2 (2.0)	1 (0.3)
Nephrologist	2 (0.5)	2 (2.0)	0
Another specialist^§^	6 (1.5)	0	6 (2.0)
Do not know/cannot remember	5 (1.2)	1 (1.0)	4 (1.3)

All data are reported as numbers and percentages of patients.

† The first HCP consulted was responsible for the diagnosis of iron deficiency, treatment initiation and prescription of medication for iron deficiency.

‡ Another specialist included an endocrinologist or rheumatologist.

§ Another specialist included a pharmacist, pulmonologist, immunologist, endocrinologist, rheumatologist or otolaryngologist.

¶ p < 0.05.

GP: General practitioner; HCP: Healthcare practitioner; ID: Iron deficiency; N/A: Not available.

Of all HCPs consulted for an initial consultation, GPs were most often consulted by all respondents (57.7%) and were the preferred HCP for females (68.5%) and younger-aged (18–25 years) respondents (75.8%), whereas males more often consulted a hematologist (39.2%) than any other HCP (Table 3). At diagnosis of ID, more females than males were informed by their HCP about symptoms (54.0 vs 42.2%), how to manage ID (32.8 vs 11.8%), consequences of poor treatment compliance (22.8 vs 13.7%), and dietary changes (23.8 vs 9.8%). Moreover, more females than males reported being prescribed ferritin and hemoglobin tests to confirm the diagnosis of ID (ferritin: 66.8 vs 32.4%; hemoglobin: 58.9 vs 46.1%), whereas more males than females were prescribed a complete blood cell count (65.7 vs 45.4%).

### Treatment of ID

GPs were the most common HCP type to initiate ID treatment in females (63.6%) and in younger-aged respondents (18–25 years; 71.2%), while hematologists were most often responsible for treatment initiation in males (46.1%) (Table 3). Most respondents (89.9%) had blood tests performed after the start of their ID treatment; around a third (32.5%) underwent blood testing within the first 3 months of ID treatment, and most underwent follow-up blood testing every 2–3 months (29.0%) or 4–6 months (39.6%). Around three-quarters (73.5%) of all 404 respondents reported that they still had ID despite treatment; half (54.2%) of 297 respondents had been diagnosed more than 4 years earlier than this survey (n = 107 did not complete this question).

### Current ID status

Of the 297 respondents with current ID, fatigue, weakness and tiredness remained the predominant symptoms for around two-thirds (65.3%) of respondents, while around a third (36.4%) complained of headaches. Of the 284 respondents who reported discomfort with their current symptoms, 76.8% considered these symptoms bothersome (20.1% really bothersome and 56.7% somewhat bothersome). Of the 297 respondents who reported which HCP they were currently consulting for ID treatment, GPs were most often cited (55.6%), followed by hematologists (24.2%), and gastroenterologists (6.7%).

### Treatment satisfaction & compliance

Satisfaction ratings for iron medications used currently or in the past 12 months ranged between 7.0 and 7.7 out of 10.

Slightly more than half of all respondents (56.4%) stayed on ID treatment for as long as prescribed (Table 4). There was no difference between males and females (52.0 vs 58.0%), but fewer than half of patients aged 18–25 years (45.0%) completed the treatment course as prescribed. Around a third (30.4%) took their medication for almost as long as prescribed. Almost two-thirds (58.9%) took their medication every day as prescribed, and another a third (34.9%) took their medication almost every day; very few respondents missed taking most or almost all tablets as prescribed (Table 4). An overall 41.1% of all respondents were not fully compliant with tablet intake. Some respondents (10.1%) stopped their medication when they felt better, while 2.5% frequently forgot to take it and therefore stopped completely. Overall, 43.6% of respondents did not comply with the prescribed duration of treatment. Among 176 respondents who cited reasons for their noncompliance (Table 4), around two-thirds (64.8%) said they felt much better; around one-fifth (20.5%) said they had trouble tolerating the medication.

**Table 4.** Compliance with iron deficiency treatment.

	All study participants (n = 404)
Compliance^†^	n = 404
Medication taken as long as prescribed	228 (56.4)
Medication taken almost as long as prescribed	123 (30.4)
Medication stopped when feeling better (less time than prescribed)	41 (10.1)
Medication stopped as it was frequently forgotten	10 (2.5)
Medication stopped after a few days	2 (0.5)
Daily compliance	n = 404
Medication taken every day (no tablet missed)	238 (58.9)
Medication taken almost every day (only a few tablets missed)	141 (34.9)
Medication missed quite often (most tablets missed)	20 (5.0)
Medication missed almost every day (almost all tablets missed)	5 (1.2)
Reasons for noncompliance	n = 176
I was feeling much better	114 (64.8)
My prescription was no longer valid	36 (20.5)
I had trouble tolerating my medication	36 (20.5)
I did not see any improvement	29 (16.5)
I don't want to take a treatment every day or for a long period of time	14 (8.0)
I forget to take them (spontaneous)	5 (2.8)
Other	3 (1.7)

All data are reported as numbers and percentages of patients.

† Compliance related to whether treatment was taken for the prescribed duration.

### Iron deficiency recurrence

Regarding recurrence of ID and actions taken, 75.0% of all 404 respondents reportedly suffered from more than one ID episode since the time they were diagnosed, with almost half (47.0%) stating that they had experienced two or three ID episodes, while almost one-fifth (17.1%) reported that they had experienced more than six ID episodes. Actions taken in response to symptom recurrence were most commonly a physician consultation (GP or specialist; 68.6% of 303 respondents to this question), followed by seeking of web-based information (23.1%) (Table 5). ID recurrences (≥1 or >3 recurrences) were reported more commonly in females than in males, in older- versus younger-aged individuals, among people in full-time versus those in part-time employment, and in those with low monthly incomes (€500–1499 vs €3500 per month and higher) (Table 5). During recurrent episodes, the main causes contributing to ID included insufficient dietary intake of iron (46.9% of respondents with ≥1 recurrence and 43.4% with >3 recurrences) and heavy menstrual bleeding (32.0 and 41.6%, respectively). Respondents more commonly consulted a GP or Specialist (68.6% of respondents with ≥1 recurrence and 78.8% with >3 recurrences) than family, friends or colleagues (16.8 and 9.7%, respectively) for help in resolving recurrent ID episodes. Analyses of the 113 respondents who reported having more than three ID episodes revealed that 63.7% took their medication for as long as prescribed. Many of these individuals searched for more information beyond what they were given by their HCP regarding medication-related adverse events, and they sought recommendations on ideal timing of treatment, including with or without food. Around one-fourth (26.8%) of the 113 respondents said they had trouble tolerating their medication; a similar proportion reported not wanting to take treatment every day or for a long period of time.

**Table 5.** Demographic and clinical characteristics and behaviors of respondents by level of iron deficiency recurrence.

		All respondents, by recurrence status since diagnosis		
		No recurrence (n = 101)	≥1 recurrence (n = 303)	>3 recurrences (n = 113)
Sex	Female	76 (75.2)	226 (74.6)	97 (85.8)
	Male	25 (24.8)	77 (25.4)	16 (14.2)
Age, years^†^	18–25	25 (24.8)	41 (13.5)	15 (13.3)
	45–54	29 (28.7)	101 (33.3)	44 (38.9)
Pregnancy	Yes	15 (19.7)	18 (8.0)	2 (2.1)
Health status	Good health	77 (76.2)	214 (70.9)	72 (63.7)
	Neither good nor poor health status	21 (20.8)	66 (21.9)	28 (24.8)
	Poor health	3 (3.0)	22 (7.3)	13 (11.5)
Employment status	Full-time	62 (61.4)	164 (54.1)	52 (46.0)
	Part-time (<32 h/week)	7 (6.9)	48 (15.8)	22 (19.5)
Monthly income, Euro	<499	6 (5.9)	26 (8.6)	15 (13.3)
	500–1499	14 (13.9)	66 (21.8)	32 (28.3)
	3500–4499	23 (22.8)	44 (14.5)	7 (6.2)
	4500–5499	10 (9.9)	20 (6.6)	3 (2.7)
Health issues (in prior 2 years)	Respiratory diseases	18 (17.8)	84 (27.7)	32 (28.3)
	Depression	20 (19.8)	102 (33.7)	41 (36.3)
	Stress/anxiety	54 (53.5)	202 (66.7)	90 (79.6)
	Fatigue/tiredness	60 (59.4)	219 (72.3)	95 (84.1)
	GI problems	37 (36.6)	155 (51.2)	67 (59.3)
Spontaneous awareness of brand of ID treatment	FerroGrad™	15 (14.9)	68 (22.4)	41 (36.3)
	Tardyfer™	3 (3.0)	23 (7.6)	14 (12.4)
Taking medication to treat health issues	Yes, currently for high BP	6 (30.0)	39 (50.0)	19 (65.5)
	Yes, currently for heart disease	2 (22.2)	15 (65.2)	0
	Yes, currently for depression	4 (20.0)	47 (46.1)	16 (39.0)
	Yes, currently for GI problems	16 (43.2)	65 (41.9)	24 (35.8)
Discovery of ID	At the hospital because I wasn't feeling well	3 (3.0)	20 (6.6)	12 (10.6)
First ID symptoms	Fatigue/weakness/tiredness	67 (66.3)	220 (72.6)	98 (85.0)
	Paleness	37 (36.6)	102 (33.7)	50 (44.2)
	Hair loss	24 (23.8)	96 (31.7)	42 (37.2)
	Heart palpitations	20 (19.8)	91 (30.0)	40 (35.4)
	GI disturbances	13 (12.9)	59 (19.5)	28 (24.8)
Triggers to seek help	Fatigue/weakness/tiredness	59 (61.5)	206 (69.6)	88 (79.3)
	Paleness	16 (16.7)	50 (16.9)	27 (24.3)
	Hair loss	8 (8.3)	49 (16.6)	20 (18.0)
	Shortness of breath	6 (6.3)	36 (12.2)	17 (15.3)
Main causes contributing to ID	Insufficient dietary intake of iron	68 (67.3)	142 (46.9)	49 (43.4)
	Heavy periods	22 (21.8)	97 (32.0)	47 (41.6)
	Ulcerative colitis	10 (9.9)	35 (11.6)	3 (2.7)
First action taken to treat ID	Physician consulted (GP or specialist)	74 (73.3)	211 (69.6)	94 (83.2)
	Sought advice from family/relatives/friends/colleagues	6 (5.9)	31 (10.2)	5 (4.4)
Time between first symptoms and initial consultation	1–3 months	48 (47.5)	122 (40.3)	32 (28.3)
	>1 year	7 (6.9)	27 (8.9)	17 (15.0)
First HCP consulted	GP	56 (55.4)	177 (58.4)	75 (66.4)
	Hematologist	20 (19.8)	60 (19.8)	15 (13.3)
HCP in charge of the diagnosis	GP	51 (50.5)	150 (49.5)	67 (59.3)
	Dietician	5 (5.0)	6 (2.0)	0
HCP in charge of treatment initiation	GP	56 (55.4)	173 (57.1)	73 (64.6)
	Gastroenterologist	10 (9.9)	17 (5.6)	3 (2.7)
HCP currently seen to treat ID	GP	40 (58.0)	125 (54.8)	62 (64.6)
	Hematologist	18 (26.1)	54 (23.7)	16 (16.7)
HCP in charge of treatment prescription	GP	55 (54.5)	175 (57.8)	75 (66.4)
Knows that ID can be a recurrent issue	Yes	90 (89.1)	290 (95.7)	109 (96.5)
Duration of treatment	Medication taken almost as long as prescribed	32 (31.7)	91 (30.0)	26 (23.0)
	Medication stopped when feeling better (less time than prescribed)	5 (5.0)	36 (11.9)	11 (9.7)
	Medication stopped after a few days	0	2 (0.7)	2 (1.8)
Search for information additional to that provided by the HCP	Adverse events	39 (38.6)	154 (50.8)	63 (55.8)
	Quality of ingredients	28 (27.7)	82 (27.1)	18 (15.9)
	Source of iron	22 (21.8)	57 (18.8)	14 (12.4)
	When to take it, e.g. morning vs evening	16 (15.8)	68 (22.4)	39 (34.5)
	With vs without food	16 (15.8)	58 (19.1)	32 (28.3)
Unmet needs (TOM)	Diet change	17 (16.8)	42 (13.9)	8 (7.1)
	GI problems	4 (4.0)	28 (9.2)	19 (16.8)
	Recurrence	1 (1.0)	8 (2.6)	5 (4.4)
Reasons for non-compliance	My prescription was no longer valid	5 (13.2)	31 (22.5)	2 (4.9)
	I was feeling much better	24 (63.2)	90 (65.2)	21 (51.2)
	I had trouble tolerating my medication	10 (26.3)	26 (18.8)	11 (26.8)
	I do not want to take a treatment every day or over a long period of time	3 (7.9)	11 (8.0)	7 (17.1)
	I forget to take them	0	5 (3.6)	3 (7.3)
Lab tests requested to confirm diagnosis	RBC count	43 (46.2)	149 (51.6)	62 (59.0)
	Hemoglobinemia	36 (38.7)	176 (10.9)	71 (67.6)
	Ferritinemia	41 (44.1)	179 (61.9)	78 (74.3)
	Serum iron	16 (17.2)	87 (30.1)	40 (38.1)
Timepoint of first follow-up blood test performed after treatment start	2 months	33 (37.9)	54 (19.6)	14 (14.0)
	≥6 months	3 (3.4)	39 (14.1)	24 (24.0)
Blood test frequency	Monthly	8 (7.9)	9 (3.0)	2 (1.8)
	Every 4–6 months	28 (27.8)	132 (43.6)	49 (43.4)
	Annually	16 (15.8)	63 (20.8)	33 (29.2)
	I only had one blood test after the start of treatment	8 (7.9)	6 (2.0)	2 (1.8)
Information provided by HCP	Description of symptoms of ID	52 (51.5)	154 (50.8)	68 (60.2)
	Cause/origin of my ID	33 (32.7)	142 (46.9)	51 (45.1)
	Consequences of not taking ID treatments	13 (12.9)	70 (23.1)	30 (26.5)
	How to improve and manage ID	21 (20.8)	90 (29.7)	30 (26.5)
	Changes in my diet/lifestyle to better cope with ID	16 (15.8)	66 (21.8)	33 (29.2)
	None	0	2 (0.7)	2 (1.8)
Actions taken when symptoms reoccurred	Physician consulted (GP or specialist)	–	208 (68.6)	89 (78.8)
	Sought Internet information	–	70 (23.1)	11 (9.7)
	Sought advice from family/relatives/friends/colleagues	–	51 (16.8)	11 (9.7)
	Sought advice from pharmacy	–	41 (13.5)	9 (8.0)
	Sought advice from paramedical professional	–	37 (12.2)	3 (2.7)
	Directly purchased a product	–	27 (8.9)	15 (13.3)
Current symptoms	Fatigue/weakness/tiredness	35 (50.7)	159 (69.7)	80 (83.3)
	Irritability or depressive mood	9 (13.0)	56 (24.6)	24 (25.0)
	Shortness of breath	8 (11.6)	52 (22.8)	24 (25.0)
	Heart palpitations	6 (8.7)	48 (21.1)	20 (20.8)
	Difficulties in concentrating	5 (7.2)	44 (19.3)	19 (19.8)
	GI disturbances	4 (5.8)	24 (10.5)	19 (19.8)
Compliance	Medication taken every day, no tablet missed	71 (70.3)	167 (55.1)	58 (51.3)
	Not fully compliant with treatment intake	30 (29.7)	136 (44.9)	55 (48.7)
Tools that would facilitate patient compliance	Pill reminder in a digital health application	44 (43.6)	111 (36.6)	28 (24.8)
	Good tolerability/does not cause GI problems	40 (39.6)	162 (53.5)	79 (69.9)
	Getting rid of all ID-related symptoms	29 (28.7)	123 (40.6)	61 (54.0)

All data are reported as numbers and percentages of patients.

† Predefined study quotas on age specified a good distribution across age groups for females and a focus for males on those aged 45–80 years. However, when it was discovered that the in-depth interviews in Part 2 were excluding quite a significant number of males, the survey was revised to include males aged 25 years and over.

BP: Blood pressure; GI: Gastrointestinal; HCP: Healthcare practitioner; ID: Iron deficiency; TOM: Top of mind.

Among all respondents, medication compliance became increasingly less common with increases in the number of recurrent ID episodes; for example, the proportion of respondents taking their medication every day was 70.3% among respondents who had no ID recurrence but 51.3% among respondents who had ≥3 episodes of recurrence (Table 5). Similarly, among respondents with ID recurrence, fewer were interested in using a pill reminder in a digital health application than having a treatment that offers better tolerability, fewer gastrointestinal (GI) problems, and is capable of resolving all ID-related symptoms (Table 5).

## Discussion

This online survey study reveals insights into the awareness, diagnosis and treatment behaviors among patients with ID in Italy from the patient’s perspective. Results reveal patient profiles in relation to diagnosis (including the type of HCPs consulted), disease knowledge, treatment satisfaction, treatment compliance and ID recurrence, which will help HCPs in Italy better personalize their management of patients with ID.

Overall, the findings from this study indicate that the respondents were largely unaware of their ID status prior to diagnosis, which was usually during a routine check-up, rather than as a consequence of respondents consulting specifically for symptoms. High prevalence rates of IDA and IDWA have also been found in research conducted among Portuguese adults aged ≥18 years [9] and nonpregnant females aged 12–21 years in the United States [10], suggesting that many individuals are unaware of their diagnosis (although neither study ascertained patient awareness of ID status). Certainly, the authors of one of these studies concluded that better education is needed for individuals at higher risk of developing ID [9]. Similarly, other studies have observed a low awareness of anemia among patients with chronic kidney disease (CKD), suggesting that they would benefit from education about anemia and its management in CKD [11,12]. However, patient education is only part of the problem; underdiagnosis may also occur lack of clinician awareness in IDWA [1] and neglected clinical signs and symptoms (especially fatigue, which is nonspecific) in IDA [2]. It has been suggested that raising awareness of IDWA among clinicians, especially in primary care, may reduce the prevalence of undiagnosed IDWA [1]. An online survey of European patients with inflammatory bowel disease (IBD) and ID, and specialists managing patients with IBD, found differences between patients and physicians in what they considered to be the predominant symptoms of ID [8]. Consistent with our results, patients reported weakness and tiredness as the key symptoms, along with paleness, whereas physicians identified paleness, breathlessness and dizziness as the primary symptoms [8]. In this survey, 60% of patients with ID secondary to IBD experienced lengthy delays (>1 year) between the onset of symptoms and ID diagnosis [8]. Even longer delays (average of 2.9 years) between symptom onset and diagnosis and treatment have been reported by patients with heavy menstrual bleeding and IDA [7]. Some researchers have suggested the importance of screening initiatives to facilitate early diagnosis and treatment of ID [9,10]. The European Hematology Association (EHA) guidelines recommend screening individuals at risk of ID, including any menstruating girl or woman of reproductive age [13].

Most respondents consulted a GP as their first HCP in seeking treatment for ID (57.7% of respondents), with similar results reported in a 2018 Mexican study, although the proportion was much higher in the Mexican study (83.7% of patients) [14]. Hematologists were much less commonly consulted (16.3% in the Mexican study [14] and 19.8% in our survey). Differences in study design, study populations and national medical systems may account for any differences in proportions.

Our survey findings also suggest that sex has more discriminatory value than age in regard to ID management, with clear differences observed between males and females in reporting of symptoms, triggers leading to the first action taken to treat ID, and the time taken between symptoms and initial action. Moreover, sex-related differences were observed as to the first HCP consulted, subsequent referrals, laboratory tests conducted for confirmation of ID status. More males than females discovered their ID status after experiencing symptoms; females more commonly discovered their ID status after a routine check-up. This is likely due at least in part to different approaches to screening between men and women. ID is common in apparently healthy women [15], as reflected in the EHA recommendations to screen all menstruating females [13], whereas screening for ID in males is limited to those at risk including athletes, vegetarians, elderly people and those with chronic illness or a history of gastric surgery [13]. At each step of the ID diagnosis and treatment pathway, the main HCP consulted by females was the GP, whereas males tended to more often consult a hematologist and a gastroenterologist to a lesser extent. This may in part reflect the fact that males at risk of ID include those with chronic illness [13]. However, it may also reflect differences in health-seeking behavior between men and women [16]. For example, similar marked discrepancies have been found in research that has explored GP use in general (not specifically ID populations), where results of one investigation into healthcare service utilization and costs in Sweden found that men were more likely than women to receive specialist inpatient care, and that women were more likely to receive primary care (more assessable, less expensive) [17]. The Swedish study concluded that it is necessary to eliminate barriers preventing men from investing in their health and seeking primary care [17]. Recent Australian research echoes this sentiment, with the finding that men are less likely than women to engage with GP health services (although it is important to note that the GP visits in this analysis only reflect health service use, not diagnosis, health condition or health service need) [18]. Some of the differences in health-seeking behavior between the sexes is driven by male perceptions of masculinity. Qualitative research has identified an attitude among some males that a ‘real man’ does not seek help for small health complaints [19], and that males do not want to waste the GP's time with what they perceive to be trivial concerns [19,20]. Instead, males prefer to seek medical help only when the symptoms prevent them from doing something [19]. Thus, male patients may be more likely to end up under the care of a specialist because their condition has progressed from a minor complaint to something more serious.

Global recommendations on ID management advise clinicians to treat ID regardless of whether or not anemia is involved [1]. The importance of replenishing iron stores (usually via oral or parenteral administration) is well-recognized, as it aims to restore hemoglobin levels to normal, and improves quality of life, physical performance, morbidity, cognitive function, work productivity, prognosis in chronic disease and pregnancy-related outcomes [1,3,5,21,22]. In this survey, treatment was prescribed by the HCP responsible for ID diagnosis; the most commonly used treatment schedule was long-term, intermittent use of oral iron; daily continuous treatment was less common.

Of concern is the finding that around two in five respondents did not take their prescribed medication for as long as directed. Many of them (around two-thirds) stated that they felt better before the end of the prescribed treatment period. Patients need to understand that a prescription must be followed through to the end, or risk worsening their ID. Among the 113 respondents who had more than three ID episodes, 36.3% did not take their medication for as long as prescribed. We did not conduct any formal correlation analyses, but this seems to suggest a relationship between stopping treatment early and recurrence rate. The HCP can play a key role in supporting patients on prescribed treatment, and explain why it is important to take the treatment for its entire duration. When asked what tools would facilitate patient compliance with ID medication, women stated that medication should be easier to tolerate with fewer GI problems, and be capable of resolving all ID-related symptoms; younger-aged women also expressed interest in receiving better information at the time of prescription, whereas males favored a pill reminder in a digital health application.

Oral iron formulations are recommended for iron replenishment [23], because they are inexpensive, show good bioavailability, are available in multiple preparations, and can effectively replenish iron stores and correct anemia [3]. However, oral iron preparations are commonly limited by GI adverse events such as constipation, diarrhea, nausea and vomiting, which have been associated with poor adherence and consequently inadequate treatment outcomes [1,3]. These adverse events are partly reflected in the results of this survey, and thus underline the importance of providing information and support for people on ID treatment, who need education about how to deal with GI adverse events and how to better manage their disease.

Around half (54.2%) of all respondents in our study were diagnosed more than 4 years ago and as many as three-quarters (73.5%) of all respondents reported they still had ID at the time of this survey, with the same symptoms as those described at initial diagnosis. Notably, over half (57.0%) of all respondents described their ID symptoms as ‘somewhat bothersome’. These findings may suggest a lack of follow-up and re-evaluation of patients by their HCPs, which was also an observation made in the above-mentioned Mexican study [14].

A number of limitations to this study should be acknowledged. By their nature, cross-sectional survey studies are prone to selection bias, sampling bias, nonresponse bias and recall bias. In addition, because we collected patients’ experiences that were not linked to clinical information, we cannot be certain about the accuracy of diagnosis. For example, our definition of ID recurrence was based on the patient’s experience of ID symptom recurrence, and did not require laboratory confirmation. A lack of data in this study regarding details of iron therapies administered to the study participants (including use of intravenous iron), details about Hb status, quality of life, how iron therapy affected prognosis in chronic disease and pregnancy-related outcomes, and treatment-related details about any adverse outcomes associated with oral iron preparations, prevent any comparisons of the study findings with the international literature. However, the strengths of this study include the collection of a large array of data variables relating to the ID care pathway and the valuable insights derived from obtaining the patient’s perspective on their experience and behaviors in relation to ID. These insights can help to improve patient care and follow-up.

## Conclusion

This survey study identified both a high prevalence of self-reported ID symptoms and recurrent ID episodes among respondents. Regardless of sex, ID symptoms were mainly generic, consisting of fatigue, weakness and tiredness, headaches and pale complexion, and were regarded as quite bothersome. Satisfaction with ID treatment was rated as average, which may in part relate to the fact that only slightly more than half of all respondents stated they took their treatment for as long as prescribed, with no differences between males and females. The predominant reason given by respondents for partly or completely stopping treatment was because they were feeling much better.

The study results highlight the importance of increasing the awareness of ID and its consequences among both HCPs and individuals in the general population, in order to shorten the time before diagnosis, and raise awareness about disease management. It is important that HCPs provide information for individuals on ID treatment, so that they better understand the management of the disease. When people are well educated and feel supported around their treatment, tolerability and treatment persistence generally improve. In particular, HCPs can play a vital role around informing their patients of the importance of continuing treatment for many months to resaturate serum iron levels, even if they are feeling better after starting treatment.

## Summary points

- The limited real-world data on the management of patients with iron deficiency anemia/iron deficiency without anemia (IDA/IDWA) in Italy indicate that ID is undertreated and treatment compliance is poor, making it a public health issue.
- This 2023 online survey study sought to identify and describe, from the patient’s perspective, the care pathways used to manage IDA/IDWA in Italy, by collecting information on how, when, and by whom patients were diagnosed, their sources of information and influence, and their knowledge of the disease.
- The questions explored patients’ perceptions and fears surrounding treatment, any treatment-related concerns, unmet needs and expectations and underlying causes of treatment compliance and persistence.
- The findings reveal a high prevalence of self-reported ID symptoms and recurrent ID episodes among respondents, and that only 56% of respondents take their treatment for as long as prescribed (the proportion is even lower [45%] among people aged 18–25 years), putting them at risk of worsening ID.
- Moreover, the survey respondents revealed a lack of knowledge of the disease and underestimation of symptoms, and a low awareness of ID among their healthcare practitioners (HCPs).
- It is crucial that HCPs and individuals in the general population become more aware of ID and its consequences, so that patients are diagnosed earlier and commence appropriate treatment, and experience better long-term consequences (reduced recurrence and progression).
- HCPs can provide a vital role for patients with ID by creating a positive experience with ID treatment through the development of educational material about ID, and supporting patients around their treatment, and advising how to minimize gastrointestinal adverse events so that patients may be motivated to stay on treatment for as long as needed to resaturate serum iron levels, instead of stopping when they feel better.
- Finally, the survey findings suggest ways in which tools would facilitate treatment compliance: men are eager to have a pill reminder in a digital health application, while women want better tolerability and fewer gastrointestinal problems with ID treatment, a higher number of tablets per box and treatment that resolves all ID-related symptoms.

## Supplementary Material


