# Supplementary material for: Iron deficiency treatment pathway in Italy: patients’ perceptions of diagnosis and treatment
Source: J Comp Eff Res. 2025 Nov 28;14(12):e250011. doi: 10.57264/cer-2025-0011 (PMC12679649; doi:10.57264/cer-2025-0011)
Supplement: Supplementary file 1 [file cer-14-250011-s1.docx]

**Supplementary Materials**

## **Supplementary Appendix 1**

### ***Survey questionnaire***

Questionnaire on iron deficiency patient care pathway administered by APLUSA

**SCREENING QUESTIONS**

**Before we start the interview, we need to ask you some preliminary questions, to confirm that you are the most relevant person to take part in our survey.**

1. **Are you…?**

Female

Male

1. **To which of the following age groups do you belong to?**

Under 18 yrs

18-25 yrs

26-44 yrs

45-54 yrs

55-80 yrs

>80 yrs

1. **Are you currently pregnant?**

Yes

No

Prefer not to answer

1. **Do you have any child(ren) (under 18 years old)?**

Yes

No

1. **How many child(ren) aged under 18 years old do you have?**

**|__|**

1. **How old are your child(ren) under 18 years old?**

Child 1 Child 2 Child 3 . . .

|_____| y.o. |_____| y.o. |_____| y.o. |

1. **In the last 2 years, did you suffer from any of the health issues below?**

|  | **Yes** | **No** |
| --- | --- | --- |
| Diabetes |  |  |
| High blood pressure |  |  |
| High cholesterol |  |  |
| Heart disease |  |  |
| Respiratory Diseases (Asthma, Shortness of breath, Chronic cough) |  |  |
| Depression |  |  |
| Stress/anxiety |  |  |
| Iron deficiency or iron deficiency anemia |  |  |
| Fatigue/tiredness |  |  |
| Constipation |  |  |
| Gastrointestinal problems |  |  |
| Cancer |  |  |

1. **Have you been taking any medication to treat the health issues below?**

|  | **Yes,** within the last 12 months | **Yes,** currently | **No** |
| --- | --- | --- | --- |
| Diabetes |  |  |  |
| High blood pressure |  |  |  |
| High cholesterol |  |  |  |
| Heart disease |  |  |  |
| Respiratory Diseases (Asthma, Shortness of breath, Chronic cough) |  |  |  |
| Depression |  |  |  |
| Stress/anxiety |  |  |  |
| Iron deficiency or iron deficiency anemia |  |  |  |
| Fatigue/tiredness |  |  |  |
| Constipation |  |  |  |
| Gastrointestinal problems |  |  |  |
| Cancer |  |  |  |

1. **You mention that you suffer(ed) from iron deficiency or iron deficiency anemia. Has this issue been diagnosed by a healthcare professional, meaning he/she told you you had ‘iron deficiency or iron deficiency anemia’.**

Yes

No

1. **Please specify all the products that you are aware of for the treatment of iron deficiency or iron deficiency anemia.**

*Please enter one product per line.*

I am not aware of any products

1. **Please select the statement that best describes how familiar you are with each of the following iron deficiency treatments.**

|  | I am not aware of this treatment | I am aware of this treatment but I have never taken it | I have already taken this treatment more than 12 months ago | I have already taken this treatment within the last 12 months | I am currently taking this treatment |
| --- | --- | --- | --- | --- | --- |
| Cromatonferro |  |  |  |  |  |
| Ferlixit |  |  |  |  |  |
| Ferritin |  |  |  |  |  |
| Ferro Complex |  |  |  |  |  |
| Ferrogyn |  |  |  |  |  |
| Ferro gluc.EG |  |  |  |  |  |
| FerroGrad |  |  |  |  |  |
| Niferex |  |  |  |  |  |
| Glucoferro |  |  |  |  |  |
| Intrafer |  |  |  |  |  |
| Losferron |  |  |  |  |  |
| Sideral |  |  |  |  |  |
| Tardyfer |  |  |  |  |  |
| Tecnofer |  |  |  |  |  |
| Iron supplements without prescription |  |  |  |  |  |
| Other, please specify |  |  |  |  |  |

1. **The treatment you are currently taking or took over the last 12 months for your iron deficiency or iron deficiency anemia has been ...**

|  | **Treatments currently used** | | | **Treatments used in the last 12 months** | | |  |
| --- | --- | --- | --- | --- | --- | --- | --- |
| …Prescribed by a healthcare …professional |  |  |  |  |  |  | |
| …Recommended by a …pharmacist |  |  |  |  |  |  | |
| …Purchased by you without further recommendation |  |  |  |  |  |  | |

**PATIENT’S PATHWAY**

**From now on, we will be focusing on your iron deficiency or iron deficiency anemia. It will be referred as ‘iron deficiency’ in the questionnaire.**

1. **Looking back to the start, how did you find out that you were suffering from iron deficiency?**

I was experiencing symptoms, so I looked for a solution

It was discovered during a check-up routine

It was discovered during pregnancy blood tests (Women only)

It was discovered at the hospital when I was admitted because I was not feeling well

Other, please specify

1. **At the time of this discovery or in the weeks preceding it, did you suffer from any of the symptoms below?**

Fatigue/ weakness/tiredness

Headaches

Paleness

Hair loss

Irritability or depressive mood

Shortness of breath

Heart palpitations (fast-beating, fluttering or pounding heart)

Difficulties in concentrating

Gastrointestinal disturbances

Other, please specify

**No, I had no symptoms**

1. **What specific symptoms led you to seek help to treat your iron deficiency issue?**

Fatigue/weakness/tiredness

Headaches

Paleness

Hair loss

Irritability or depressive mood

Shortness of breath

Heart palpitations (fast-beating, fluttering or pounding heart)

Difficulties in concentrating

Gastrointestinal disturbances

Other, please specify

1. **How would you describe the symptoms you experienced at that time of diagnosis?**

Really bothersome

Somewhat bothersome

Neither bothersome nor not

Not so bothersome

Not bothersome at all

1. **What would you say has caused your iron deficiency?**

Insufficient dietary intake of iron

Pregnancy (Women only)

Menopause (Women only)

Heavy periods (Women only)

Ulcerative colitis

Following bariatric surgery

Fibroids

Renal impairment

Digestive/gastro disorders

A treatment for another disease/health problem

Other, please specify

1. **What action did you take first when seeking help to treat your iron deficiency issue?**

I consulted a physician (General practitioner or specialist)

I sought information on internet

I asked for advice to family/ relatives/ friends/ colleagues

I went straight to the pharmacy to get advice

I consulted/ asked for advice to a paramedical professional (physiotherapist, nurse, midwife)

I sought advice from a specialized store (food supplements stores, phytotherapy stores, …)

I directly purchased a product for my problem

I adjusted my lifestyle / diet

Other, please specify

1. **Thinking about the initial consultation you had with a healthcare professional where iron deficiency was first brought up by you or him. How long after the first signs did this happen?**

**Less than a month** after experiencing the symptoms

Between **1 to 3 months** after experiencing the symptoms

Between **4 to 6 months** after experiencing the symptoms

Between **7 to 12** months after experiencing the symptoms

**More than a year** after experiencing the symptoms

1. **Which healthcare professional did you see at first to treat these symptoms?**

General practitioner

Gastroenterologist

Hematologist

Gynecologist

Oncologist

Nephrologist

Dietician

Nurse

Midwife

Pharmacist

Another specialist, please specify:

Don’t know / cannot remember

1. **Were you referred to another healthcare professional for further exams at this time? If so, which one:**

General practitioner / Another General practitioner

Gastroenterologist / Another Gastroenterologist

Hematologist / Another Hematologist

Gynecologist / Another Gynecologist

Oncologist / Another Oncologist

Nephrologist / Another Nephrologist

Dietician / Another Dietician

Nurse / Another Nurse

Midwife / Another Midwife

Pharmacist / Another Pharmacist

Another specialist: _______________________

I have **not been referred** to any healthcare professional

Don’t know / cannot remember

**Q10. Which healthcare professional made the diagnosis of iron deficiency?**

General practitioner

Gastroenterologist

Hematologist

Gynecologist

Oncologist

Nephrologist

Dietician

Nurse

Midwife

Pharmacist

Another specialist: _______________________

Don’t know / cannot remember

**Q11. When was the diagnosis done?**

**Year: I________I**

**Q12. Did the healthcare professional request you to do lab tests to confirm the diagnosis of iron deficiency? If yes, which one(s)?**

Red blood cell count

Complete blood cell count

Hemoglobinemia

Ferritinemia

Serum iron

Other, please specify

**No lab tests were requested**

**I do not remember**

**Q13. Was a blood test performed after the start of treatment?**

Yes

No

**Q14. How long after the start of treatment?**

1 month

2 months

3 months

4 months

5 months

6 months or more

**Q15. How often do you have blood tests to check your iron level?**

Every month

Every 2 or 3 months

Every 4 to 6 months

Once a year

Less than once a year

Other, please specify

**I only had one blood test after the start of treatment**

**Q16. When you were diagnosed as having iron deficiency what information did your healthcare professional provide you with?**

Description of symptoms of iron deficiency

Severity of my iron deficiency (mild, moderate, severe)

Cause/origin of my iron deficiency

Treatment options available

Potential side effects

Consequences of not taking iron deficiency treatments

Possible complications of iron deficiency

How to improve and manage iron deficiency

Changes in my diet/lifestyle to better cope with iron deficiency

Other, please specify

**Q17. Which healthcare professional prescribed the first treatment for iron deficiency?**

General practitioner

Gastroenterologist

Hematologist

Gynecologist

Oncologist

Nephrologist

Another specialist: _______________________

Don’t know / cannot remember

**Q18. Since you have been diagnosed with iron deficiency for the first time, which among the situations below best describes your personal situation?**

I had this issue of iron deficiency only **once**

I had this issue of iron deficiency **2 or 3 times**

I had this issue of iron deficiency **4 to 6 times**

I had this issue of iron deficiency **more than 6 times**

**Q19. When your symptoms re-occurred, what did you do?**

I consulted a physician (General practitioner or Specialist)

I sought information on the Internet

I asked for advice from family/relatives/friends/colleagues

I went straight to the pharmacy to get advice

I consulted/ asked for advice from a paramedical professional (physiotherapist, nurse, midwife)

I sought advice from a specialized store (food supplements stores, phytotherapy stores, …)

I directly purchased a product for my problem

I adjusted my lifestyle/diet

Other, please specify

**I didn’t do anything**

**Q20. Are you still suffering from iron deficiency?**

Yes

No

**Q21. Which of the symptoms below are you still presenting today?**

Fatigue/weakness/tiredness

Headaches

Paleness

Hair loss

Irritability or depressive mood

Shortness of breath

Heart palpitations (fast-beating, fluttering, or pounding heart)

Difficulties in concentrating

Gastrointestinal disturbances

Other, please specify

**No, I have no symptoms**

**Q22. How would you describe the symptoms you experience?**

Really bothersome

Somewhat bothersome

Neither bothersome nor not

Not so bothersome

Not bothersome at all

**Q23. Which healthcare professional are you currently seeing to treat your iron deficiency?**

General practitioner

Gastroenterologist

Hematologist

Gynecologist

Oncologist

Nephrologist

Dietician

Another specialist: _______________________

**No one – I do not see a healthcare professional for iron deficiency any more**

Don’t know / cannot remember

**Q24. Did you know that iron deficiency could be a recurrent issue, meaning that it can occur often/repeatedly?**

Yes

No

**Q25. Has the healthcare professional in charge of the follow-up of your iron deficiency informed you about potential relapses and what to do if symptoms reoccurred?**

Yes

No

**EXPERIENCE AND SATISFACTION TOWARDS TREATMENTS**

**Looking back to the treatment you are currently taking for your iron deficiency or that you took over the last 12 months.**

**Q26. Which healthcare professional prescribed this treatment to you?**

General practitioner

Gastroenterologist

Hematologist

Gynecologist

Oncologist

Nephrologist

Another specialist: _______________________

Don’t know / cannot remember

**Q27. Could you please specify what was the duration of treatment prescribed (time on the script written by your physician)?**

**Q28. And for how long did you take this treatment, or have you been taking it?**

|  | **Duration of treatment initially prescribed** | | | |  | **Duration of intake** | | | |
| --- | --- | --- | --- | --- | --- | --- | --- | --- | --- |
|  | **Less than 2 months** | **Between 2 to 3 months** | **Between 4 to 6 months** | **More than 6 months** |  | **Less than 2 months** | **Between 2 to 3 months** | **Between 4 to 6 months** | **More than 6 months** |
| **Treatments currently used** | | | | | | | | | |
|  |  |  |  |  |  |  |  |  |  |
|  |  |  |  |  |  |  |  |  |  |
|  |  |  |  |  |  |  |  |  |  |
|  |  |  |  |  |  |  |  |  |  |
|  |  |  |  |  |  |  |  |  |  |
| **Treatments used in the last 12 months** | | | | | | | | | |
|  |  |  |  |  |  |  |  |  |  |
|  |  |  |  |  |  |  |  |  |  |
|  |  |  |  |  |  |  |  |  |  |
|  |  |  |  |  |  |  |  |  |  |
|  |  |  |  |  |  |  |  |  |  |

**Q29. Regarding the treatment you are currently taking for your iron deficiency or that you took over the last 12 months, which sentence best reflects your behavior?**

I take/took my iron deficiency medication every day, I do/did not miss any tablet

I take/took my iron deficiency medication almost every day, I only miss/ed my tablet occasionally (a few times per month)

I forget/forgot to take my tablets quite often (several times a week)

I have/had a lot of trouble taking my medication, I forget it almost every day

**Q30. Regarding the duration of treatment, which sentence best reflects your behavior?**

I take/took my iron deficiency medication as long as prescribed by my physician

I take/took my iron deficiency medication almost as long as prescribed by my physician

I stop/stopped taking my iron deficiency medication as soon as I feel/felt better (less time than recommended by my physician)

I stop /stopped taking my iron deficiency medication because I was tired of taking it and frequently forgot it (less time than recommended by my physician)

I take/took my iron deficiency medication only for a few days and stopped

**Q31. Why haven’t you followed your healthcare professional recommendation in terms of treatment duration?**

My prescription was no longer valid

I was feeling much better

I did not see any improvement

I had trouble tolerating my medication

I don't want to take a treatment every day or over a long period of time

Other please specify

**Q32. Have you ever discussed with your physician about the fact that…?**

Yes No

…You missed iron deficiency tablets

…You did not take your iron deficiency

tablets as long as recommended

**Q33. How satisfied are you with the iron medication you are currently taking or took over the last 12 months?**

*Please use a scale from 1 to 10, where:*

*1 = Not at all satisfied*

*10 = Extremely satisfied*

|  | **1**  **Not at all satisfied** | **2** | **3** | **4** | **5** | **6** | **7** | **8** | **9** | **10**  **Extremely satisfied** |
| --- | --- | --- | --- | --- | --- | --- | --- | --- | --- | --- |
| **Treatments currently used** |  |  |  |  |  |  |  |  |  |  |
| **Treatments used in the last 12 months** |  |  |  |  |  |  |  |  |  |  |

**Q34. Could you please evaluate the iron medication you are currently taking or you took over the last 12 months on the criteria below?**

*Please use a scale from 1 to 10, where:*

*1 = Does not apply at all*

*10 = Totally applies*

|  | **Treatments currently used** | | | **Treatments used in the last 12 months** | | |
| --- | --- | --- | --- | --- | --- | --- |
| Is effective in reducing symptoms related to iron deficiency | __/10 | __/10 | __/10 | __/10 | __/10 | __/10 |
| Works rapidly | __/10 | __/10 | __/10 | __/10 | __/10 | __/10 |
| Does not cause gastrointestinal problems | __/10 | __/10 | __/10 | __/10 | __/10 | __/10 |
| Is easy to find at the pharmacy | __/10 | __/10 | __/10 | __/10 | __/10 | __/10 |
| Is affordable | __/10 | __/10 | __/10 | __/10 | __/10 | __/10 |

**Q35. In addition to the information provided by the healthcare professional, did you look for information about the iron deficiency medication prescribed? If so, what type of information did you look for?**

Efficacy

Side effects

Quality of ingredients

Source of iron

Dosing

Frequency

Duration of treatment

When to take it, e.g. morning vs evening

With vs without food

**I didn’t look for any information on the treatment prescribed**

**Q36. Besides iron medication, were you recommended any lifestyle changes and or specific diet?**

Yes

No

**REACTIONS TOWARDS TOOLS / INFORMATION**

**Q37. What could make it easier for you to take your iron treatment as recommended by your healthcare professional?**

**Better information when prescribed or delivered**

**Helpful packaging**

Pill reminder in a digital health application

Blister pack with calendar

**Convenience**

Less frequent intake compared to the current treatment

Ability to take the medicine without water

Higher number of tablets per box (for example 90 tablets)

**Tolerability**

Good tolerability/ Does not cause gastrointestinal problems

**Efficacy**

Getting rid of all symptoms related to iron deficiency

**Other, please specify**

**Q38. Overall, what are the key challenges you face with your iron deficiency treatments that you would like to change?**

**DEMOGRAPHIC**

**Q39. What is your overall health status?**

Very good

Rather good

Neither good nor poor

Rather poor

Very poor

**Q40. What region do you live in?**

Central

Islands

Northeast

Northwest

South

**Q41. Would you say the area you live in is…**

Rural

Urban (within city limits)

Suburban (outside of city limits)

Small city/town

**Q42. What is your current employment status?**

Unemployed/looking for work

Employed full time

Employed part-time (less than 32 hours)

Stays home to care for family

Retired/disabled/not working and not looking for work

Non-paid work (e.g., volunteer, charity)

Student/at school

**Q43. What is your monthly net household income (on average)?**

Less than 499 Euro

500–1,499 Euro

1,500–2,499 Euro

2,500–3,499 Euro

3,500–4.499 Euro

4,500–5,499 Euro

Over 5,500 Euro

Do not want to respond

Thank you for participating in and completing our research.
